# Experimental studies on the characteristics of chisel picks in coal cutting for bucket wheel excavators

**DOI:** 10.1038/s41598-024-55746-7

**Published:** 2024-03-04

**Authors:** Zhenning Su, Bo Song, Zhongxin Wang, Chang Liu, Long Sun, ZiJian Li, Mingmin Guo

**Affiliations:** 1 Digital and Intelligent Industry Center, CCTEG Shenyang Engineering Company, Shenyang, 110013 China; 2 School of Mining, Liaoning Technical University, Fuxin, 123000 China

**Keywords:** Chisel pick, Rock cutting test, Cutting force, Mechanical excavation, Coal mining, Engineering, Energy infrastructure, Mechanical engineering

## Abstract

Chisel pick is a basic and important rock cutting tool, and the performance of chisel pick directly affects rock mining. In this paper, a rock cutting device was developed for chisel picks cutting experiments. The influence of the depth of cutting, width of chisel pick, rake angle, back clearance angle and tip fillet radius on the cutting performance such as cutting force, normal force, and specific energy has been comprehensively studied. In addition to the general conclusions, the experimental results show that the back clearance angle has an influence range on the cutting, and the ratio of the normal force to the cutting force decreases with the increase of the rake angle; the tip fillet radius greatly improve the mean cutting force and specific energy. The experimental results will provide data support for the design of chisel picks on rock excavation machinery and a more reasonable chisel pick cutting rock mechanics model.

## Introduction

Chisel picks have long been used as a primary tool for cutting rocks in the field of geology and engineering, it is commonly used in bucket wheel excavators^1^ and suction dredgers^2,3^ now. In addition, there are a variety of mechanical stripping rock drag tools, such as conical pick, radial pick, PDC (polycrystalline diamond compact) etc., which are used to various types of excavation and tunnel machinery for cutting rocks of different hardness^4^. The field measurements showed that the chisel tools require 3–4 times less energy than the disc cutters in especially soft- to medium- strength rocks^5,6^. The usage of chisel tools in soft rock formations is usually standard^7^. For excavation machinery, chisel picks facilitate digging and collecting simultaneously. Chisel pick is the simplest form of rock-cutting tool, and the study of its cutting mechanism will facilitate the understanding of the rock-cutting process. Since Evans put forward the theory of tensile failure, after 60 years of research on chisel picks, people have not fully understood the rock cutting mechanism of chisel picks, and the existing theoretical models have deviations in the calculation of cutting resistance. More detailed laboratory data will help to establish a model that more accurately describes the rock cut by the chisel pick.

Many scholars have developed experimental equipment and study the cutting process of picks. Barker^8^ described a rig which designed for full-scale work on rock cutting. Allington^9^, Roxborough and Rispin^5^, Ozdemir^10^ and Fauvel^11^ developed early versions of linear rock cutting machine and carried out rock cutting experiments. Mohammadi, et al.^12^ introduced a small-scale linear cutting machine (SSLCM) at the Mechanized Excavation Laboratory of Tarbiat Modares University. This test rig is the modified Klopp shaping machine, which has a power of 5.9 kW and a maximum cutting stroke of 900 mm. SSLCM used by Rostami, et al.^13^, Bejari and Hamidii^14^. Ouyang et al.^15^ introduced a Linear Rock Cutting Test Equipment (LRCTE) comprised by a pedestal, rock clamp, cutter clamp, sliding block, set of guide rails, cutting driver, control panel, measurement system, and set of drag picks. LRCTE can provide a thrust force as large as 100 kN on the drag pick with a cutting speed that ranges from 1 to 10 mm/s. The maximum cutting depth is 20 mm, and the maximum cutting width is 20 mm. The cutting length each time can be as large as 160 mm. Yasar and Yilmaz^16,17^ designed Vertical Rock Cutting Rig (VRCR) as a mobile test equipment used by any hydraulic press. Rock cutting speed ranges between 0.5 and 1 cm/s, and data recording speed is 50 data/s by VRCR. 10 × 23 × 20 cm^3^ can be cut in VRCR. Tumac, et al.^18^ introduced a new generation of portable linear rock cutting machine (PLCM) developed in the Mining Engineering Department of Istanbul Technical University. 20 × 20 × 10 cm^3^ block samples can be cut in PLCM, cutting resistance has a precision in the order of 1 kN and covering a range from 0 to 100 kN^19^. Dogruoz, et al.^4^ introduced a rock cutting test setup which mainly consists of a planer, a dynamometer and a data recording unit. The modified planer has a stroke of 625 mm and a power of 4 kW. The setup can accommodate a block of rock having a length of 500 mm, a width of 350 mm and a height of 300 mm. Copur^20^ introduced a full-scale linear rock-cutting machine used in chain saw machines full scale chisel tool with the real cutting conditions.

On the basis of the development of experimental equipment, a large number of chisel pick experiments were carried out to study the effects of rock strength^4,21^, mineral composition^18^, water content^14^, cutting depth^15,22^, pick width^6,15^, rake angle^23,24^, wear^4^ and other factors on cutting force, normal force, specific energy and chip particle size. In previous experiments, rocks with medium hardness were mainly cut, most of the tool rake angles used were about 0 degree, and most of the cutting depths were less than 10 mm. This is inconsistent with the parameter value range of the chisel pick of the coal mining bucket wheel excavator. And there are no experimental studies on back clearance angle and tip fillet, which are important for the design of chisel picks.

In terms of the theoretical model of chisel cutting rock, Evans^25^ established the theoretical model of chisel cutting rock based on the rock tensile failure criterion, and then the application range was extended to blunt wedges^26^ and point attack-picks^27^. Nishimatsu^28^ established a theoretical model of chisel cutting rock based on the shear failure criterion. Xue et al.^29^ considered the effects of rock compact core and normal force, and used the torque balance of the Evans’ model to give the expression of horizontal cutting force and vertical propulsive force. Miedema^30^ divided rock failure, according to brittle-ductile failure and tensile-shear failure, into: flow type, tear type, chip type, shear type and crushed type. The failure type is determined according to the ratio of rock UCS and BTS, and the Evans, Nishimatsu and Merchant Model is used to describe different failure types and calculate the cutting force. Ouyang, et al.^31^ considered the three-dimensional geometry of the chip and the crushed zone of the tool tip, established the crushed zone expansion induced tensile (CEIT) failure model, and proposed a simplified form for easy application. Some research results show that the theoretical models might overestimate or underestimate cutting force and cannot give reliable results^22^. Although the chisel pick is a more basic shape of cutting tool, it has been less experimentally and theoretically studied than the conical pick.

The range of cutting depth and rake angle of soft rock excavation equipment, such as bucket wheel excavator and cutter suction dredger, is larger than the existing experimental range^30,32^. And the real pick tip is rounded, which is different from the shape of the tool used in the existing experiments. It is necessary to conduct chisel pick experiments with a larger range of geometric parameters to guide the establishment of theory and engineering practice.

In this paper, a chisel picks cutting experiment is carried out for a bucket wheel excavator mining coal. A device for linear rock cutting based on a universal testing machine is developed. On the basis of the new device, a comprehensive experimental study is carried out on the influence of the depth of cutting, width of chisel pick, rake angle, back clearance angle and tip fillet radius on the cutting force, normal force and specific energy. Experimental results are discussed and compared with theoretical model predictions.

## Rock cutting theory models for chisel picks

### Evans tensile failure model

**Figure 1:**
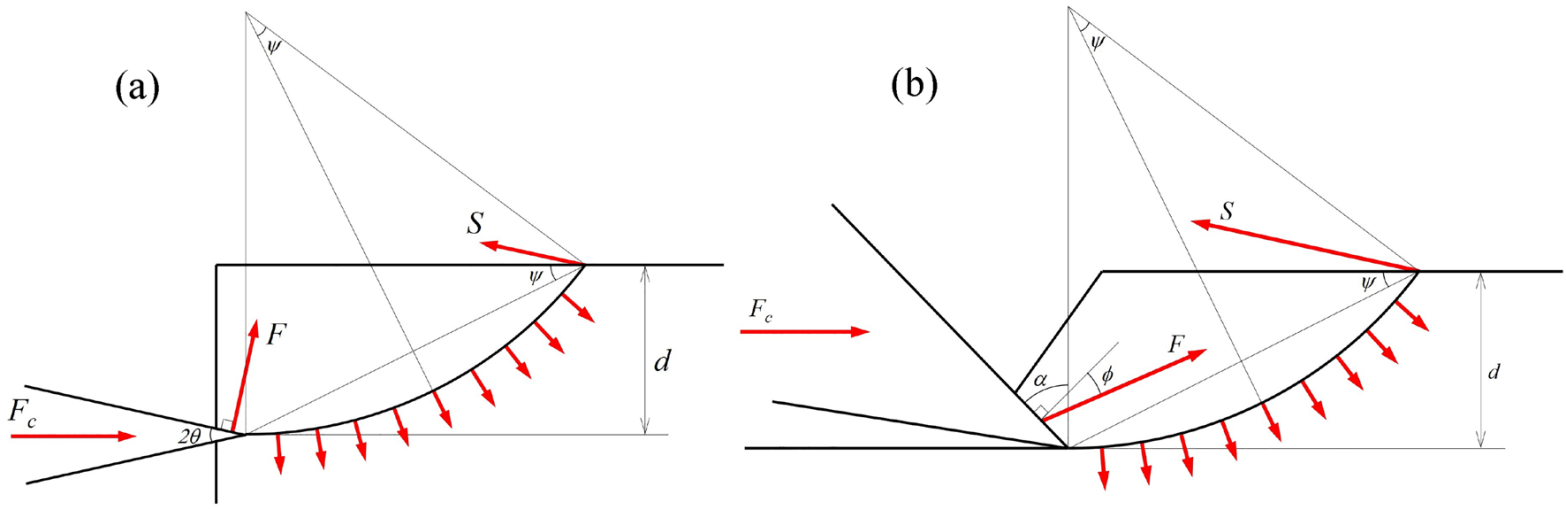
Rock cutting tensile failure model with chisel pick. (**a)** Evans model. (**b)** Improved Evans model.

Evans^25^ proposed the first theoretical model of rock cutting based on the Merchant metal cutting model. The Evans model considered a symmetrical chisel pressed into coal, and the cracks propagated to the free surface in the form of an arc, as shown in Fig. 1a. The model takes the intersection of the crack and the free surface as the center of rotation, and establishes the moment balance between the vertical force on the front surface of the chisel pick and the tensile stress on the fracture surface. The functional relationship between the cutting force *F*_c_ and the breakthrough angle *Ψ* established by the moment balance. The angle of breakage that minimizes the cutting force is calculated based on the differential of the cutting force on the breakout angle is equal to 0. Finally, the cutting force formula for the chisel pick is:

1 $$F_{c} = \frac{{2\sigma_{t} wd\sin \theta }}{1 - \sin \theta }$$

where, *σ*_*t*_ is the rock tensile strength, *w* is the tool width, *d* is the cutting depth, and *θ* is the tool half-cone angle. If the friction between the front surface of the tool and the rock is considered, replace *θ* with *θ* + *ϕ*, and *ϕ* is the friction angle between the tool and the rock.

The Evans model is based on the chisel tool penetration, and the back surface of the chisel tool hinders the continuous cutting of the rock. Roxborough^33^ modified the Evans model, and an asymmetrical chisel tool cutting rock formulation was proposed, as shown in Fig. 1b. Positive back clearance angle of asymmetrical chisel tools are not considered due to wear effects. *π*/2−*α* is the chisel pick wedge angle, (*π*/2−*α*)/2 replaces *θ* in the original formula, and considering friction, the tensile failure formula actually applied is:

2 $$F_{c} = \frac{{2\sigma_{t} wd\sin [(\pi /2 - \alpha )/2 + \phi ]}}{1 - \sin [(\pi /2 - \alpha )/2 + \phi ]}$$

### Nishimatsu shear failure model

**Figure 2:**
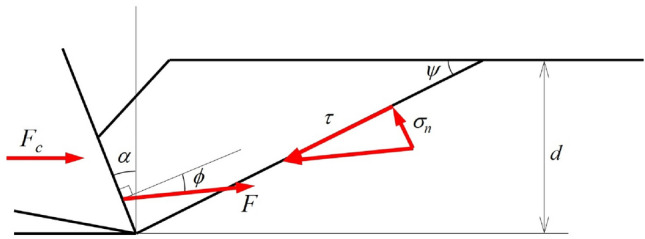
Nishimatsu rock cutting shear failure model.

Nishimatsu^28^ proposed a theoretical model of rock cutting based on shear failure, as shown in Fig. 2. The Nishimatsu model assumes that the normal stress on the rock fracture surface decreases exponentially from the chisel tip to the free surface. In the limit state on the fracture surface, the shear stress and normal stress conform to the Mohr–Coulomb shear failure criterion:

3 $$\tau = c + \sigma_{n} \tan \varphi$$

where *τ* is the shear stress, *σ*_*n*_ is the normal stress, *c* is the cohesion and *φ* is the internal friction angle. The functional relationship between the cutting force *F*_c_ and the breakthrough angle *Ψ* established by the force balance. Similar to the Evans model, the angle of breakage that minimizes the cutting force is calculated based on the differential of the cutting force on the breakout angle is equal to 0. The cutting force formula of the Nishimatsu model is:

4 $$F_{c} = \frac{2cwd\cos \varphi \cos (\phi - \alpha )}{{(n + 1)[1 - \sin (\phi - \alpha + \varphi )]}}$$

where *n* is the stress distribution factor, fitted according to the experiment *n* = 11.3−0.18*α* and the unit of the rake angle *α* is degree.

### Crushed zone expansion induced tensile failure model (CEIT model)

**Figure 3:**
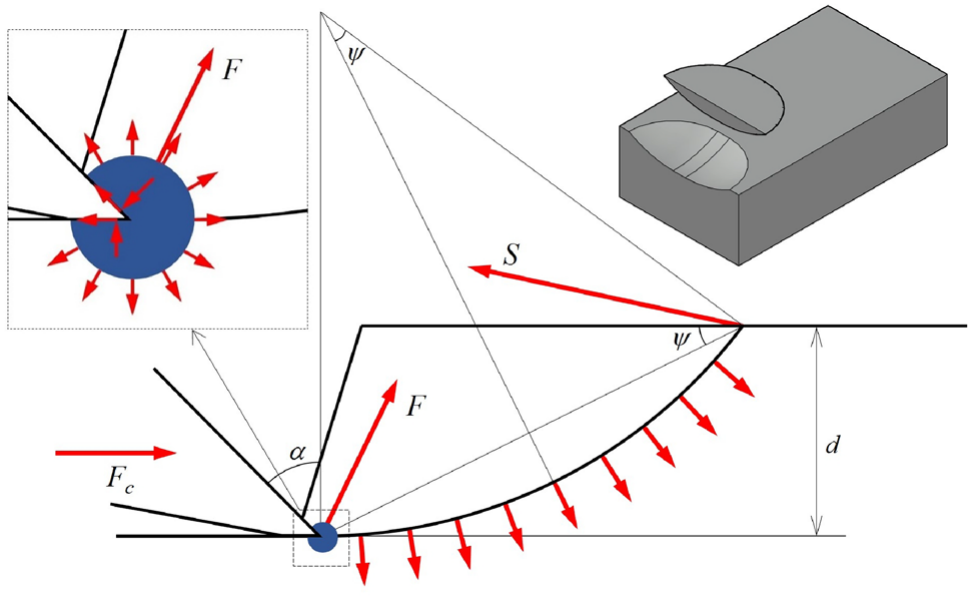
Crushed zone expansion induced tensile failure model.

Ouyang, et al.^31^ based on the Evans tensile failure model, assuming that the tip of the chisel pick has a crushed fracture zone and the fracture surface of the rock is composed of a spherical surface and a cylindrical surface, proposed crushed zone expansion induced tensile (CEIT) failure model, as shown in Fig. 3. The cutting force formula of the CEIT model is:

5 $$F_{c} = \frac{{\sigma_{t} wd[\cos \alpha + \tan \phi + {\text{sgn}} (\alpha )\sin \alpha \tan \phi ]}}{4\sin \psi \sin [(\pi /2 + \alpha )/2]\sin [(\pi /2 + \alpha )/2 - \psi ]}\left[ {1 + f(\psi ) \times \frac{d}{w}} \right]$$

where, *f*(*Ψ*) is the coefficient of the three-dimensional effect of the failure surface, calculated according to the vector integral of the tensile strength on the fracture surface:

6 $$f(\psi ) = [\sin (4\psi )\cos (2\psi ) - 4\psi \cos (2\psi ) + \pi \sin^{3} (2\psi )]/(8\sin^{4} \psi )$$

The breakout angle that minimizes the cutting force is difficult to calculate by differential Eq. (5), so Ouyang, et al.^31^ proposed a simplified calculation method for the breakage angle that minimizes the cutting force:

7 $$\psi = \left( {0.246 + \frac{0.238}{{w/d + 2.08}}} \right)\alpha + \left( {0.404 + \frac{0.521}{{w/d + 3.63}}} \right)$$

The cutting force is obtained by the breakout angle into Eq. (5).

Different from the Evans model and Nishimatsu model established under the plane stress condition, the three-dimensional effect of chisel cutting rock is included in the CEIT model. Based on the experimental data of Nuh^6^, the cutting force predicted by the CEIT model is better than that of the Evans model and the Nishimatsu model.

In addition to theoretical models, cutting forces can also be calculated through empirical models based on experimental data of chisel pick cutting. Previous studies have shown that there is not a single failure mode in the rock cutting process^30,34^, and theoretical models are generally established only based on tensile or shear failure modes, and empirical models can break through this limitation. Based on the fitting of experimental data and the understanding of the cutting process, the researchers proposed different empirical models^35–38^. However, the range of application of the empirical model is limited due to the limited data used and insufficient understanding of the failure mechanism. Although the normal force plays a key role in the wear of the pick, the normal force has received less attention than the cutting force in both theoretical and empirical models^38^.

## Rock cutting experiment with chisel pick

The purpose of this study is to investigate the influence of different factors on the cutting performance of bucket wheel excavator chisel picks for cutting coal. Bucket wheel excavators are used in open pit mining for coal or overburden sand and soft rock. The compressive strength of mined rock is recommended not to exceed 15 MPa and limited to 20 MPa^39^. The cutting depth of a chisel pick changes continuously during the mining process. Based on the design of a bucket wheel excavator with a production capacity of 3900 m^3^/h, the maximum cutting depth reaches 180 mm. The rake angle, back clearance angle and shape of bucket wheel excavator chisel picks can be designed. The calculation accuracy of existing theoretical methods cannot serve the design of chisel picks for bucket wheel excavators. And the parameter range of previous experiments could not cover the target parameters, so the chisel pick cutting coal experiment was carried out.

### The properties of coal sample

The experimental coal sample was mined with an electric shovel excavator from a thick coal seam in an open-pit coal mine in eastern Mongolia, China. Large lumps of coal with a size of more than 1 × 1 × 0.5 m^3^ are screened and cut into 20 × 20 × 35 cm^3^ blocks through a stone cutter. Samples were sealed using plastic wrap as soon as possible after being cut, to prevent from being weathered.

The sample collected at the same time completed the basic physical and mechanical parameter test according to the standard. Sample density is 1.33 g/cm^3^, uniaxial compressive strength is 15.6 MPa, brazilian tensile strength is 0.89 MPa, cohesion is 1.06 MPa, internal friction angle is 32.6°, coefficient of friction with the tool is 0.4, Young's modulus is 325 MPa, Poisson's ratio is 0.12.

**Figure 4:**
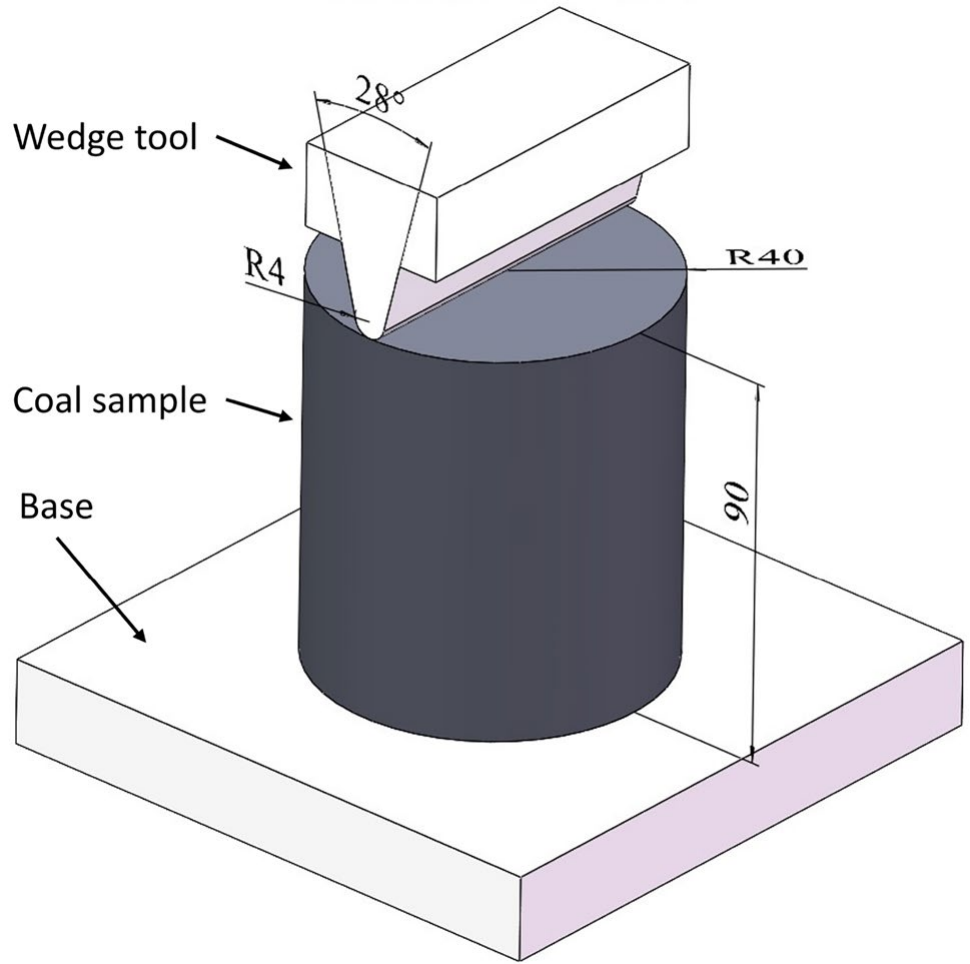
Tool and specimen dimensions of wedge test.

And a wedge test was carried out to estimate the diggability characteristic of coal by bucket wheel excavator, test equipment is shown in Fig. 4.^40^ The cutting resistance per wedge cutter length of the sample is 121.3 kN/m, which is used for the selection of bucket wheel excavators.

### Coal cutting test equipment based on electronic universal testing machine

**Figure 5:**
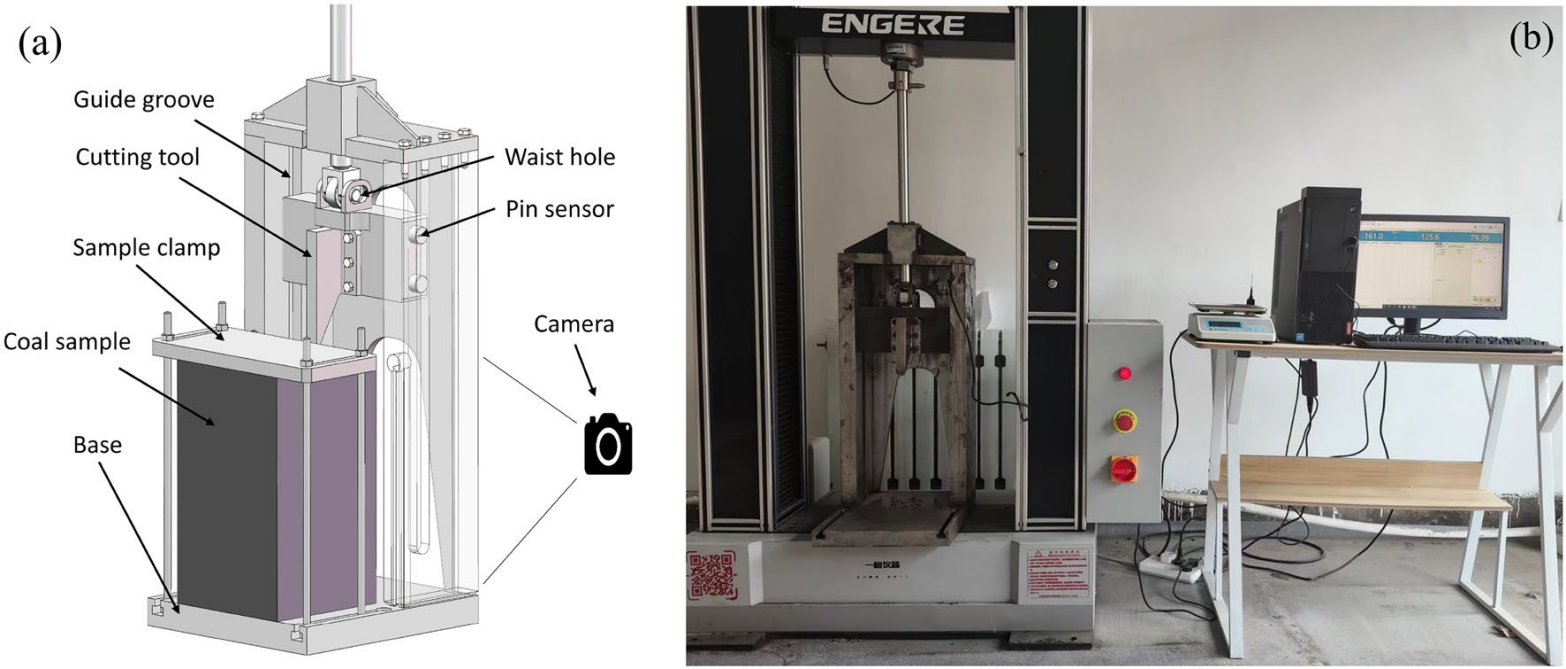
Rock cutting test machine (**a**) 3D model (**b**) actual image.

Figure 5 shows the experimental equipment combined with an electronic universal testing machine, which is used for coal cutting experiments. The coal sample is cut vertically due to the structure of the universal testing machine. Therefore, a vertical guide groove is designed to transfer the horizontal force (normal force) generated during the cutting process to the base. The vertical loading connection adopts the form of scroll wheel and slotted hole, which avoids the transmission of horizontal force to the beam. The normal force is measured by a pin force sensor moving in the guide groove. The cutting force is measured by a pancake load cell force sensor mounted on the beam of the universal testing machine. The force sensor range is 0–30 kN and the accuracy is ± 0.1%. The camera is used to record the cutting process with a frame rate of 60 and a resolution of 1080 × 1920. The experimental instrument scheme can be implemented by simply improving the universal testing machines that exist in a large number of geotechnical laboratories. The equipment cannot measure the side force, and the movement speed of the universal testing machine is limited, so it is difficult to carry out high-speed pick cutting experiments.

### Data processing

**Figure 6:**
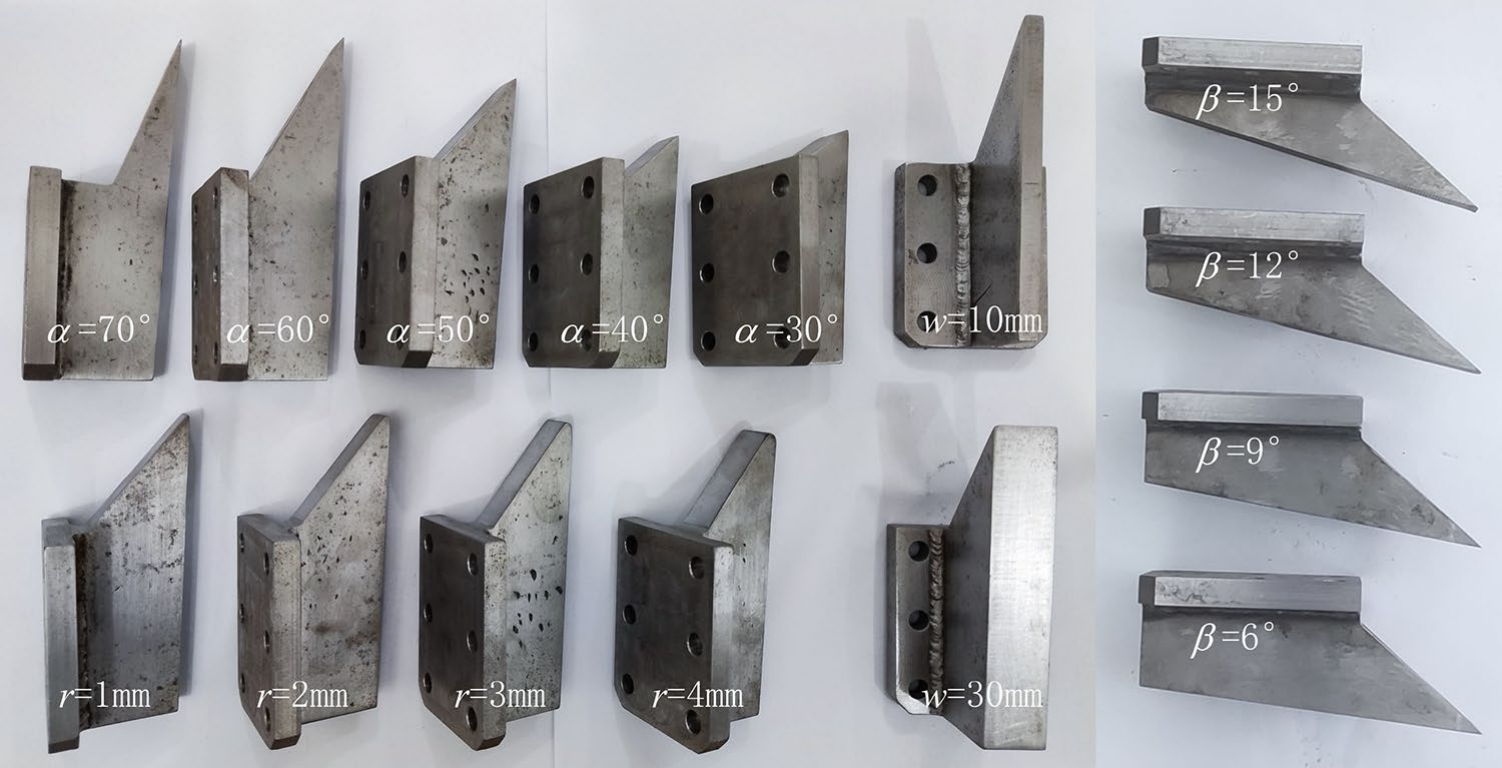
Chisel picks for test.

The experiment adopts the control variable method, and the controlled variables include: depth of cut *d*, width of chisel pick *w*, rake angle *α*, back clearance angle *β*, tip fillet radius *r*. The basic parameters of chisel teeth are *α* = 50°, *w* = 20 mm, *r* = 0 mm, *β* = 3°, on this basis, the variables are controlled. The selection of experimental parameters took into account the following factors: *d* and *w* are limited by the sample size. Larger *d* and *w* will cause both sides of the chip to exceed the plane of the sample. The values of *α* and *β* refer to the commonly used corresponding parameters of bucket wheel excavator teeth. The value of *r* takes into account the impact on *d*. The crushing zone will increase as the *r* value increases, and at the same time, the length of the chip in the *d* direction decreases. The mutual interaction between *r* and *d* will cover up the influence of *r*, so *r* needs to be much smaller than *d*. Because *d* and *w* are parameters that have been widely studied, 3 research levels are selected, while studies on *α*, *β* and *r* in the corresponding range are rare, so 5 research levels are selected. Figure 6 shows the chisel picks with different test parameters, and Table 1 shows the test parameters of each set. The cutting length is 300 mm, and the cutting speed is 500 mm/min. Each set of tests was carried out 4 times on four sides of a coal block sample, and the cutting force and normal force were measured with a frequency of 10 Hz.

**Table 1:** Cutting parameter for coal cutting test.

Index	Depth of cut, *d* (mm)	Width of chisel pick, *w* (mm)	Rake angle, *α* (degrees)	Back clearance angle, *β* (degrees)	Tip fillet radius, *r* (mm)
1	20	20	70	3	0
2	20	20	60	3	0
3	20	20	50	3	0
4	20	20	40	3	0
5	20	20	30	3	0
6	10	20	50	3	0
7	30	20	50	3	0
8	20	10	50	3	0
9	20	30	50	3	0
10	20	20	50	3	1
11	20	20	50	3	2
12	20	20	50	3	3
13	20	20	50	3	4
14	20	20	50	6	0
15	20	20	50	9	0
16	20	20	50	12	0
17	20	20	50	15	0

The peak cutting force is the maximum cutting force among four tests of the same parameter, and the peak normal force is the normal force corresponding to the peak cutting force moment. The mean cutting force and normal force are obtained by averaging the cutting force and normal force of four tests of same parameter. After the test, the peeled chips were collected and weighed using an electronic balance with an accuracy of 0.1 g to obtain the chip mass. The specific energy is calculated by the following formula:

8 $${\text{SE}} = \frac{{F_{{{\text{cm}}}} l\rho }}{Q}$$

where, *F*_cm_ is the mean cutting force of the test, *l* is the cutting length, *ρ* is density of coal, *Q* is the chip mass.

## Experimental results and discussion

During a typical cutting experiment, the cutting force and the normal force fluctuate periodically. When the large chip is peeled off, the cutting force and the normal force are at the peak. After the peeling, the cutting force and the normal force drop, and then rise fluctuatingly until the next peeling occurs.

**Figure 7:**
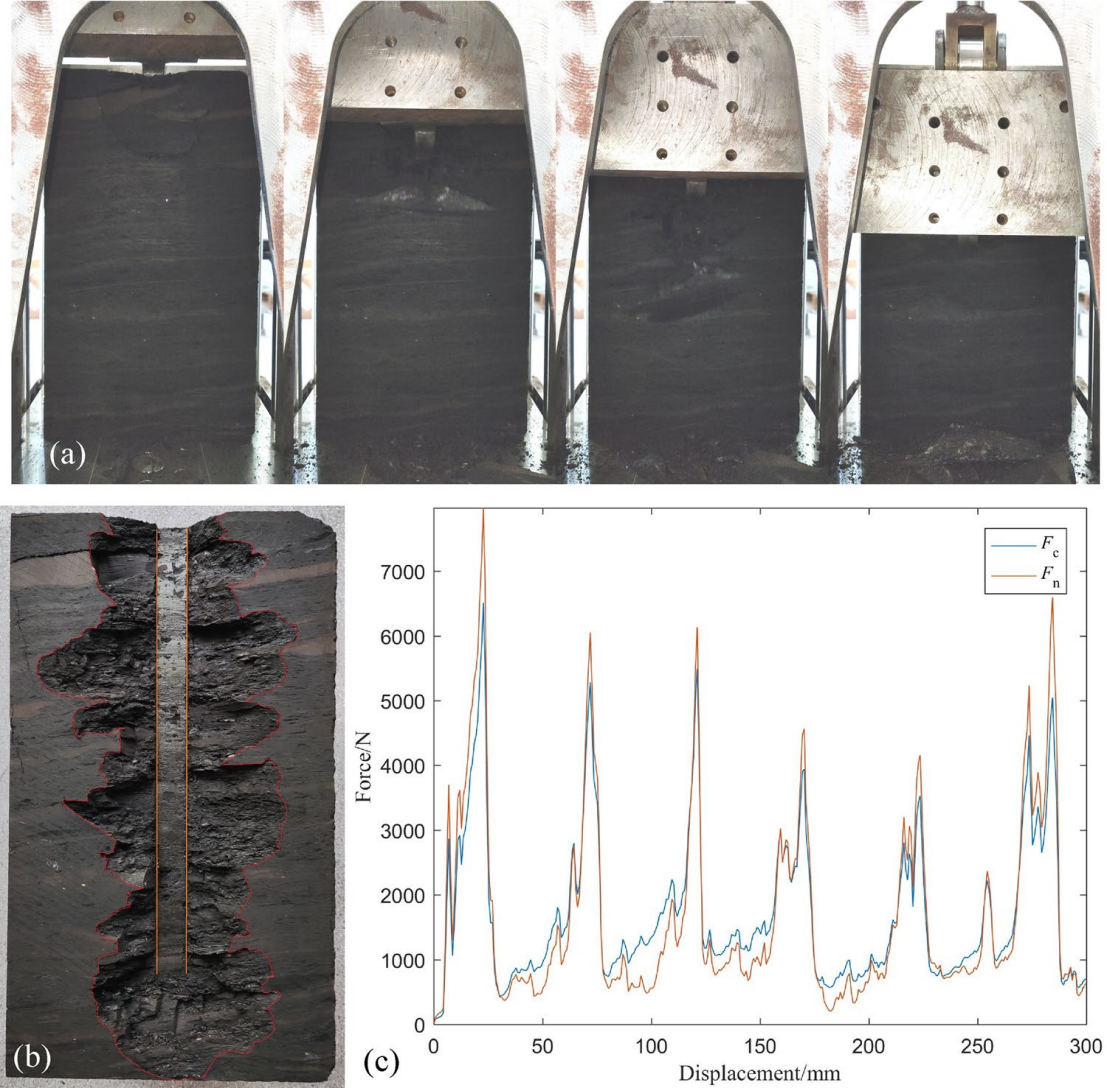
(**a**) A typical cutting test process (**b**) Cutting Groove (**c**) Cutting and normal force versus time.

**Figure 8:**
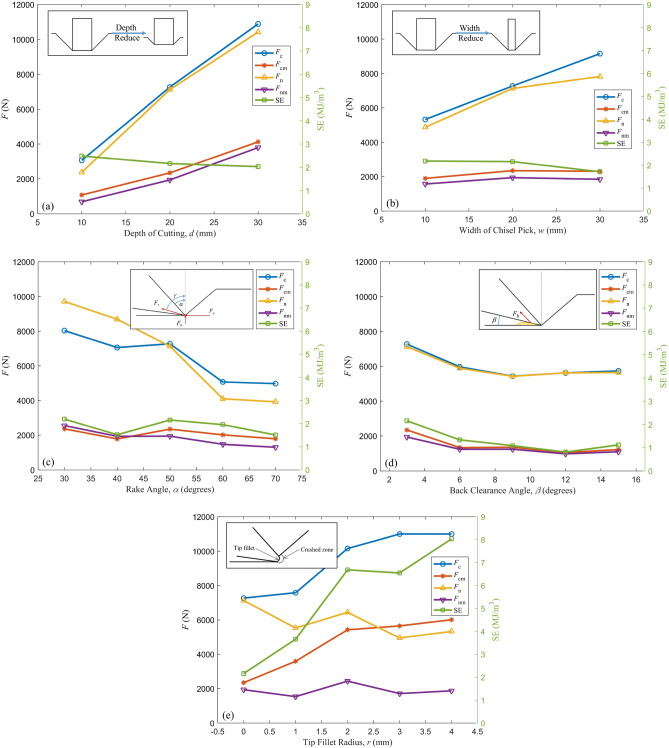
Effect of different factors on *F*_c_, *F*_cm_, *F*_n_, *F*_nm_, SE (**a**) Depth of cut (**b**) Width of chisel pick (**c**) Rake angle (**d**) Back clearance angle (**e**) Tip fillet radius.

Figure 7 shows the cutting process of a test, the typical cutting groove and the corresponding force curve. During the cutting process, the cutting force and the normal force were kept in sync. The cyclic fluctuation of the cutting force represented the phased peeling off of chips, and the two were consistent. Table 2 summarizes the results of tests with different parameters. Figure 8 shows the variation of peak cutting force *F*_c_, mean cutting force *F*_cm_, peak normal force *F*_n_, mean normal force *F*_nm_ and specific energy SE as a function of the depth of cut, width of chisel pick, rake angle, back clearance angle, and tip fillet radius, respectively.

**Table 2:** Result of coal cutting test.

Index	*F*_c_ (N)	*F*_cm_ (N)	*F*_n_ (N)	*F*_nm_ (N)	Q (g)	SE (MJ/m^3^)
1	4972	1784.6	3921	1303.1	517.8	1.503
2	5069	2011.3	4095	1468.8	423.0	1.953
3	7263	2341.2	7121	1936.1	427.6	2.155
4	7050	1775.8	8683	1926.1	477.6	1.513
5	8029	2358.8	9709	2549.2	420.3	2.194
6	3058	1077.3	2374	688.6	155.0	2.483
7	10,886	4121.9	10,420	3794.7	793.6	2.025
8	5326	1894.3	4872	1565.8	328.6	2.181
9	9148	2306.9	7819	1839.0	540.8	1.706
10	7577	3587.1	5530	1532.3	421.1	3.654
11	10,151	5424.3	6433	2434.3	330.5	6.684
12	10,992	5650.9	4947	1709.7	343.2	6.549
13	10,993	6009.1	5328	1869.0	211.5	8.035
14	5966	1334.9	5886	1240.4	386.4	1.331
15	5434	1356.3	5426	1242.0	485.8	1.083
16	5622	1049.0	5621	978.4	494.0	0.810
17	5731	1222.6	5643	1097.9	415.7	1.115

### Effects of the depth of cut and width of chisel pick

The depth of cut and width of chisel pick have similar effects on force and specific energy. With the increase of cutting depth and chisel pick width, the cutting force and normal force increase approximately linearly, and the specific energy decreases, as shown in Fig. 8a, b.

The rate of change of force with depth of cut is greater than the width of the chisel pick. The intercept of the fitting straight line of the force is approximately equal to 0 when the cutting depth is equal to 0. The difference is that the intercept is not equal to 0 when the width of the chisel pick is equal to 0. As the cutting depth approaches to 0, the cross-sectional area of the cutting groove approaches to 0. As the pick width approaches 0, the cross section of the cutting groove is approximately triangular, and cutting force is required to damage both sides of the cutting groove. This explains why the two intercepts are different.

The increase of chip volume and the increase of cutting depth have a cubic relationship based on the geometric relationship, while the cutting force and cutting depth have an approximately linear relationship in the experiment. Therefore the rate of increase in chip volume is greater than the rate of increase in cutting force, and the specific energy decreases with increasing depth of cut, based on Eq. 8. The slight decrease of the specific energy with the width of the chisel pick indicates that the destruction energy efficiency for surface within the cutting width is higher and the rock volume cut per unit energy is more when the cutting depth is 20 mm.

The resultant force of the cutting force and the normal force is the cutting resistance* F*_r_, and the angle between the direction of the cutting resistance and the normal direction is defined as $\gamma = {\text{arccot}} (F_{{\text{n}}} /F_{{\text{c}}} )$. *γ* depends upon the ratio of normal force to cutting force known as cutting coefficient. The mean cutting force and mean normal force increase simultaneously as the depth of cut and width of chisel pick increase. Because the cutting force is greater than the normal force, *γ* decreases as the depth of cut and width of chisel pick increase, and approaches 45°.

### Effect of the rake angle

Different from most chisel picks whose rake angles are near 0°, the rake angles of this experiment range from 30° to 70°. As the rake angle increases, the cutting force and normal force decrease, and the specific energy decreases.

Different from depth of cut and width of chisel pick, the specific energy reduction affected by rake angle is the result of reduced cutting force. *γ* increases from 39° to 52° while the rack angle increases from 30° to 70°. The direction of cutting resistance rotates with the front surface of the pick, but the rotation angle is smaller than the change of the rake angle. The direction of cutting resistance is located within the wedge angle of the chisel pick, which is beneficial to the force of the pick structure and reduces the damage of the pick.

### Effect of the back clearance angle

From the experimental results, when the back clearance angle is less than 6°, the cutting force and normal force increase as the back clearance angle decreases, and when the back clearance angle is greater than 6°, the cutting force and normal force are less affected by the back clearance angle, as shown in the Fig. 8d. Wang, et al.^41^ also reported in the study of conical picks that the back clearance angle affects the normal force in a small range in soft rock cutting. In the study of PDC, Gerbaud, et al.^42^ proposed that the force acting on the back of the tool constitutes a part of the cutting resistance, which is affected by the hydrostatic pressure of the tip crushed zone, rock properties, depth of cut and back clearance angle. During the cutting process, the deformation of the rock at the back of the tool and the transport of part debris of the rock from the crushed zone to the back of the tool lead to forces acting on the back of the tool. As the back clearance angle increases, the space between the back of the pick and the groove surface of the rock increases, the contact area between rock deformation and debris with the back of the pick decreases, and the pressure on the back of the pick decreases. When the back clearance angle is greater than the limit, rock deformation and debris are no longer in contact with the back of the pick, and the influence of the back clearance angle is eliminated. Therefore, the back angle has an influence range on the cutting resistance.

**Figure 9:**
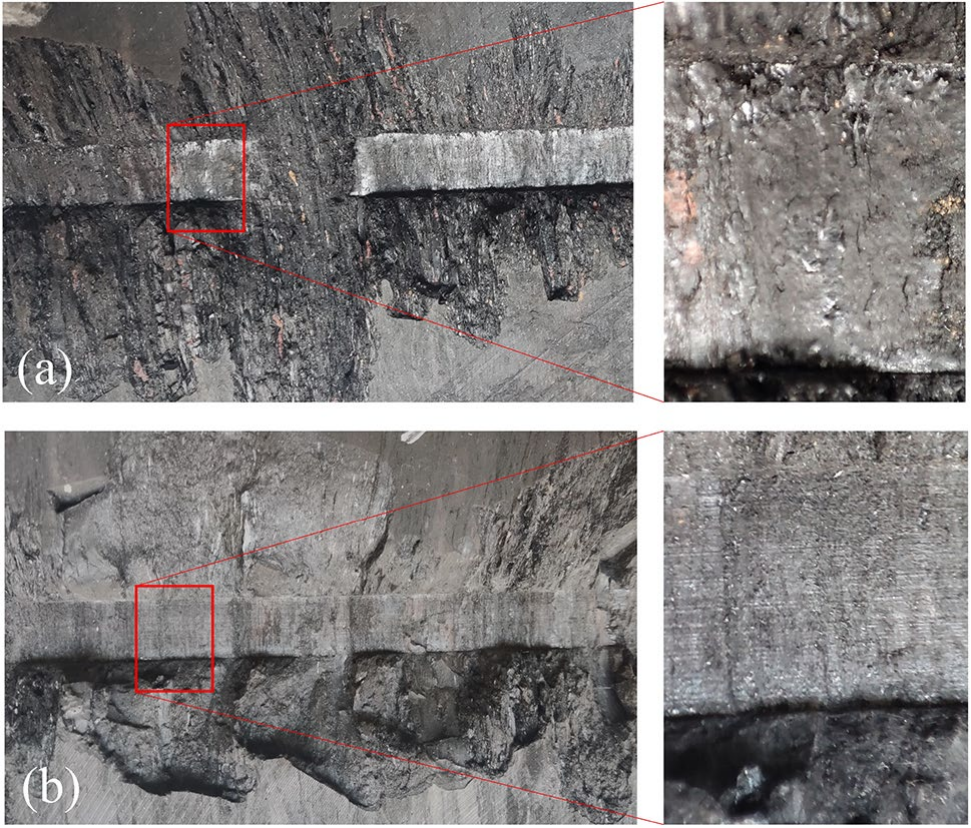
Observation of the bottom of the chisel pick groove with different back clearance angles (**a**) *β* = 3°. (**b**) *β* = 9°.

Figure 9 shows the observation of the bottom of the chisel pick groove after tests with different back clearance angles. The bottom of the groove with a back angle of 3° is smooth and has a sheet-like structure after compaction of coal debris. The bottom of the groove with a back angle of 9° is rough and has no compacted structure. This shows that the debris transported to the back of the pick are subjected to a greater force at 3°.

### Effect of the tip fillet radius

The chisel pick installed in the bucket wheel excavator is plated with tungsten carbide to reduce wear and tear, and the tip produces mm-level fillet. Meanwhile, the pre-blunt during manufacturing process prevents premature tool breakages^43^. Figure 8e shows that the peak and mean of cutting force increase synchronously with the increase of tip fillet radius. This shows that the increase of cutting force caused by tip fillet continues in the cutting process and does not fluctuate with the cutting process. This means that the rock failure caused by tip fillet is ductile failure. There is a crushing zone that increases with the radius in the area of the tip fillet. During the cutting process, the energy consumed by the crushing zone increases linearly with the size of the crushing zone. Therefore, the specific energy increases linearly with the increase of the tip fillet radius.

The influence of the tip fillet radius on the specific energy will make it difficult to define the diggability of the rock. Other parameters all change in the direction of specific energy reduction in field application, but the tip fillet radius of the chisel pick will lead to a great increase in specific energy. In this experiment, when* r* = 0 mm, the specific energy is in the range of 0.81–2.48 MJ/m^3^, as *r* increases to 4 mm, the specific energy increases to 8.04 MJ/m^3^. According to the research on the diggability of bucket wheel excavators^40^, the specific energy when *r* = 0 mm is in the "Diggable" level roughly. However, when *r* = 4 mm, the specific energy is already at the level of "Marginal" or "Undiggable".

When the cutting depth is fixed, the thickness of the ductile failure zone increases with the increase of the tip fillet radius, and the corresponding brittle failure depth decreases. Assuming that the normal force is not affected by ductile failure in the rock crushing zone, this explains the slight decrease in the peak normal force as the tip fillet radius increases.

The mean cutting force increases as the tooth tip fillet increases, and the mean normal force remains unchanged, so *γ* increases. With the pick blunted, the tip profile forms an expanding approximate tip fillet and *γ* increases. This should be noted when designing chisel picks so that the direction of *F*_r_ is within the pick wedge angle throughout the life of the pick.

### Multiple regression analysis

Multiple linear regression analysis was performed on the results of 68 tests (4 times per parameter set). Table 3 reveals the main influencing factors of *F*_c_, *F*_n_, *F*_cm_, *F*_nm_ and SE.

**Table 3:** Experimental results multiple regression analysis coefficient.

Variable	Statistics	Intercept	*d*	*w*	*α*	*β*	*r*	*R*
*F*_c_	B	48.345	360.550	127.056	−72.489	−118.558	1187.101	0.925
	S.E	1181.413	32.261	32.261	14.427	31.931	95.794	
	Beta	−	0.539	0.190	−0.242	−0.185	0.617	
	*p*-value	0.967	< 0.001	< 0.001	< 0.001	< 0.001	< 0.001	
*F*_n_	B	3390.351	362.431	103.789	−140.575	−80.244	−307.813	0.888
	S.E	1213.646	33.141	33.141	14.821	32.803	98.408	
	Beta	–	0.638	0.183	−0.553	−0.147	−0.188	
	*p*-value	0.007	< 0.001	0.003	< 0.001	0.017	0.003	
*F*_cm_	B	−461.179	152.228	20.632	−9.131	−110.918	1111.908	0.950
	S.E	698.539	19.075	19.075	8.531	18.880	56.641	
	Beta	–	0.317	0.043	−0.042	−0.241	0.804	
	*p*-value	0.512	< 0.001	0.284	0.289	< 0.001	< 0.001	
*F*_nm_	B	198.817	155.307	13.659	−29.494	−83.197	15.582	0.859
	S.E	528.519	14.432	14.432	6.454	14.285	42.855	
	Beta	–	0.699	0.061	−0.297	−0.390	0.024	
	*p*-value	0.708	< 0.001	0.348	< 0.001	< 0.001	0.717	
SE	B	3.713	−0.023	−0.024	−0.009	−0.106	1.630	0.938
	S.E	1.037	0.028	0.028	0.013	0.028	0.084	
	Beta	–	−0.036	−0.037	−0.033	−0.171	0.879	
	*p*-value	0.001	0.422	0.405	0.460	< 0.001	< 0.001	

B is the regression coefficient, S.E. is the standard error. Beta is the standardized regression coefficient. The probability value (*p*-value) is the observed level of significance for the test. The p-value is less than 0.05 (5% significance level), concluding that there is a statistically significant relationship between the measured dependent variable and the parameter at the 95% confidence level. *R* is the correlation between the measured dependent variable and the parameter.

Multiple regression analysis shows that *F*_c_ was significantly affected by all experimental parameters. The standardized regression coefficient Beta shows that *r* (0.617) and *d* (0.539) are the most influential parameters for *F*_c_. *F*_n_ is significantly affected by all experimental parameters, and *d* (0.638) and *α* (−0.553) are the most influential parameters for *F*_n_. *F*_cm_ is significantly affected by *d*, *w* and *r*, and *r* (0.804) is the most influential parameter. *F*_nm_ is significantly affected by *d*, *α* and *β*, and *d* (0.699) is the most influential factor. SE is significantly affected by *β* and *r*, and *r* (0.879) is the most influential factor.

The intercept in the *F*_n_ regression function is significant and the coefficient B is much larger than S.E.. The intercept of *F*_nm_ is not significant and its coefficient B is smaller than S.E.. Comparing the two shows that only at the moment when chip peeling occurs, there is a normal force that is not affected by other parameters. However, from the perspective of the entire cutting process, *F*_n_ does not exist naturally.

### Prediction of cutting peak force by theoretical method

Evans model, Nishimatsu model and CEIT model are used to predict the peak cutting force of No. 1–9 tests, and the results are listed in the Table 4. There are large deviations between the results and predictions of the cutting force by the three models.

**Table 4:** Comparison between the predicted value of the peak cutting force of the theoretical model and the experimental results.

Index	*F*_c_ (N)			
	Experiment	Evans	Nishimatsu	CEIT
1	4972	793.2	−1250.9	705.5
2	5069	1063.8	341.0	898.8
3	7263	1423.3	206.7	1147.3
4	7050	1915.2	177.1	1484.5
5	8029	2613.1	174.6	1976.2
6	3058	711.6	103.3	386.2
7	10,886	2134.9	310.0	2283.2
8	5326	711.6	103.3	948.5
9	9148	2134.9	310.0	1346.1

The cutting force prediction result of No. 1 test is negative by Nishimatsu model, because the rake angle is 70°, n + 1 in Eq. (4) is less than 0. Meanwhile, the Nishimatsu model has an error in the variation trend of the cutting force with the rake angle, which because the range of the rake angle in this experiment deviates from the expected range of the Nishimatsu model. Evans model and CEIT model correctly predict the changing trend of the cutting force with rake angle.

For the predicted values of numbers 6–9, the Evans model and the Nishimatsu model have the same prediction results for numbers 6/8 and 7/9. Because the Evans model and Nishimatsu model assume that the width of chisel pick and depth of cut are directly proportional to the cutting force. From the experimental results, the cutting force has only a linear relationship with the width of chisel pick, and the slope is smaller than the depth of cut. The CEIT model predicts the effect of width of chisel pick more reasonably on the cutting force by considering the three-dimensional efficiency on both sides of the groove.

In the prediction of normal force by the Evans model and the Nishimatsu model, within the front angle range of this experiment, the model predicts that the direction of normal force to the rock, which is inconsistent with the experimental results.

## Conclusions

In this paper, a series of coal cutting tests by chisel picks with different parameters were carried out in order to explore the effect of cutting parameters on force and specific energy. The following conclusions were obtained: (1) The chisel pick cutting experiment can be carried out by using the universal material testing machine through modification. (2) When the cutting depth and cutting width increase, the cutting force and normal force increase, but the specific energy decreases, the contribution of the two to the increase of cutting force is different. (3) When the current angle increases, the cutting force and normal force decrease, the resistance direction rotates with the front surface of the pick, and the change angle of the resistance direction is smaller than that of the rake angle. (4) The back clearance angle has an influence range on the cutting force. Within the influence range, when the back clearance angle decreases, the cutting force increases. There is a strong linear relationship between the tip fillet radius with the peak cutting force, the mean cutting force and specific energy. In engineering practice, using a chisel pick with a tip fillet requires more energy to break rocks.

According to the results of this experiment, CEIT model is better than Evans model than Nishimatsu model in predicting cutting force, but the prediction of cutting force of the three is much smaller than that of the experimental structure, which shows that there is still insufficient understanding of the mechanism of chisel pick cutting mechanics.

It should be noted in the results that the back clearance angle is an important geometric parameter, which is often neglected in previous studies. The normal force is an important component of the cutting resistance and should be included in the cutting mechanics model. This study helps to understand the rock cutting mechanism of the chisel pick, especially under the condition of large rake angle range and tip fillet, which is of great significance for the design of the bucket wheel excavator pick and the establishment of a more accurate mechanical model of the chisel pick cutting rock.

This study only used one type of coal as the cutting material, so the results cannot reflect the effect of rock strength on rock cutting. For the chisel pick on a wheel bucket excavator, multiple picks work simultaneously to complete the rock cutting process. Multiple picks cutting experiments are of great significance to the design of picks for rock mining equipment. In the future, more rock cutting experiments and multiple picks cutting experiments will be carried out.

## Data Availability

Data supporting the results of the study can be accessed upon reasonable request from the corresponding author.
